# Stabilization of Mixed-Anion (O^2−^/S^2−^) Networks in ZnO-Substituted Silicate–Phosphate Oxysulfide Glasses: Linking Cation–Sulfide Bonding to Thermal and Dielectric Properties

**DOI:** 10.3390/ma19040734

**Published:** 2026-02-13

**Authors:** Justyna Sułowska, Luka Pavić, Andrzej Kruk

**Affiliations:** 1 AGH University of Krakow, Faculty of Materials Science and Ceramics, al. Mickiewicza 30, 30-059 Krakow, Poland; 2 Division for Materials Chemistry, Ruđer Bošković Institute, Bijenička Cesta 54, 10000 Zagreb, Croatia; 3 AGH University of Krakow, Faculty of Space Technologies, al. Mickiewicza 30, 30-059 Krakow, Poland

**Keywords:** oxysulfide glasses, partial substitution of MgO with ZnO, sulfide bonding, DSC, X-ray absorption spectroscopy, electrochemical impedance spectroscopy

## Abstract

Mixed-anion silicate–phosphate oxysulfide glasses have attracted increasing interest due to their tunable thermal stability, electrical response, and potential use in functional glass and glass–ceramic materials. In this work, silicate–phosphate oxysulfide glasses in the SiO_2_-P_2_O_5_-K_2_O-MgO-SO_3_-ZnO system were examined to determine how partial substitution of MgO with ZnO influenced their thermal and electrical properties under reducing conditions. Melting in a strongly reducing atmosphere predominantly converted sulfur to reduced sulfur species, producing mixed oxygen–sulfur glass networks. Differential scanning calorimetry (DSC) shows that ZnO substitution reduces the configurational heat capacity at the glass transition (ΔC_p_) by up to ~40%, suppresses crystallization exotherms, and shifts crystallization onset temperatures by more than 100 °C toward higher values, indicating enhanced network rigidity. Potassium and magnesium K-edge X-ray absorption spectroscopy (XAS) revealed increased short-range ordering around Mg^2+^ in Zn-free glasses after heat treatment, whereas Zn-containing glasses remain more structurally disordered. Impedance spectroscopy demonstrated that ZnO-substituted glasses exhibit higher activation energies for electrical transport (≈0.9–1.0 eV) and lower AC conductivity compared to Zn-free compositions, reflecting restricted alkali-ion mobility. These results demonstrate that partial substitution of MgO with ZnO significantly enhances the thermal stability and electrical insulating behavior of reduced silicate–phosphate oxysulfide glasses, providing valuable structure–property insights for the design of thermally stable functional glasses and glass–ceramics.

## 1. Introduction

Glasses are non-crystalline solids that can be described as supercooled liquids lacking long-range atomic order, whereas glass–ceramics are obtained through controlled crystallization of glasses, resulting in composite materials consisting of crystalline phases embedded in a residual amorphous matrix [1]. Owing to their broad compositional flexibility, glasses and glass–ceramics enable precise tailoring of thermal, structural, and electrical properties, making them attractive for optical, dielectric, and ion-transport-related applications [2].

In recent years, increasing attention has been directed towards mixed-anion glass systems containing both oxide (O^2−^) and sulfide (S^2−^) species [3,4,5]. Compared to conventional oxide glasses, the incorporation of sulfur introduces higher anion polarisability and weaker bonding, which strongly affects thermal stability, dielectric relaxation, and crystallization behavior [3,4,5]. Therefore, such oxysulfide glasses provide a valuable platform to investigate the relationship between anion chemistry, cation coordination, and structure–property correlations [3,4,5].

Silicate–phosphate glasses constitute a particularly versatile structural framework for mixed-anion systems [6,7]. The coexistence of SiO_4_ and PO_4_ tetrahedra allows systematic control of network polymerization and non-bridging oxygen content, while the introduction of sulfur under reducing conditions leads to mixed O/S coordination environments around both network formers and modifier cations [8]. As a consequence, silicate–phosphate oxysulfide glasses often exhibit complex relaxation phenomena and pronounced sensitivity of thermal and electrical properties to compositional changes [9,10].

Due to their mixed-anion character and compositional flexibility, silicate–phosphate oxysulfide glasses are considered promising materials for applications requiring a combination of tunable thermal stability, dielectric response, and ionic transport. In particular, such glasses have been investigated as solid electrolytes and dielectric materials, as sealing glasses for high-temperature and electrochemical devices, and as precursor materials for functional glass–ceramics obtained through controlled devitrification. The presence of sulfur in the glass network enables modification of crystallization behavior and electrical relaxation processes, offering additional degrees of freedom to tailor properties relevant to these applications.

The nature of the modifier and intermediate cations plays a decisive role in determining the structural and functional behavior of these glasses [11]. These environments are associated with high polarizability and structural flexibility, which favor dipolar relaxation and a frequency-dependent dielectric response. In contrast, alkaline-earth cations such as Mg^2+^ exhibit higher field strength and preferentially form stronger Mg-O or mixed Mg-O-S links, which can promote local structural ordering and act as nucleation centers during thermal treatment [12,13].

Zinc oxide represents a particularly important component in multicomponent glass systems because Zn^2+^ can act as a network modifier or as an intermediate oxide, depending on composition and redox conditions. In oxide and oxysulfide glasses, zinc is commonly coordinated in tetrahedral ZnO_4_ or mixed ZnO_x_S_4−x_ units with a significant covalent contribution, leading to increased bond directionality and local network rigidity [14,15,16]. Previous studies have shown that ZnO incorporation can suppress crystallization, modify glass transition behaviour, and alter dielectric relaxation by restricting long-range cation mobility and enhancing localized polarization processes [17].

Despite growing interest in mixed-anion glasses, systematic experimental correlations between K-S, Mg-S, and Zn-S bonding environments and the resulting thermal stability, crystallization behavior, and frequency-dependent dielectric properties remain limited, particularly for potassium-based silicate–phosphate oxysulfide glasses. In particular, the combined influence of ZnO substitution for MgO on glass transition behavior, devitrification pathways, and electrical relaxation mechanisms has not yet been comprehensively clarified.

With these application perspectives in mind, the aim of the present work is to systematically investigate the effect of partial substitution of MgO by ZnO on the thermal behavior, sulfur retention, crystallization tendency, local cation coordination and electrical properties of reduced silicate–phosphate oxysulfide glasses in the SiO_2_-P_2_O_5_-K_2_O-MgO-SO_3_ system. Differential scanning calorimetry (DSC), X-ray diffraction (XRD), X-ray absorption spectroscopy (XAS) at the K and Mg K-edges, and impedance spectroscopy are combined to establish structure–property relationships and to elucidate how zinc incorporation modifies local structure, devitrification behavior, and ionic transport in mixed-anion glass networks.

## 2. Experimental Procedure

### 2.1. Glass Synthesis and Composition

Two series of sulfur-containing silicate–phosphate glasses were prepared within the SiO_2_-P_2_O_5_-MgO-K_2_O-(ZnO)-SO_3_ compositional system. The first series consisted of Zn-free compositions, while the second included 5 mol% ZnO, introduced at the expense of MgO to evaluate the effect of zinc incorporation on network stability. Both series contained a constant level of modifiers (20 mol% K_2_O) and sulfur (5 mol% SO_3_), while the SiO_2_/P_2_O_5_ ratio was systematically varied to tune the degree of network polymerization. The nominal batch compositions (in mol%) were as follows:

- Zn-free glasses:
  39SiO_2_-8P_2_O_5_-28MgO-20K_2_O-5SO_3_ (labelled 39-5S);
  38SiO_2_-9P_2_O_5_-28MgO-20K_2_O-5SO_3_ (labelled 38-5S);
  37SiO_2_-10P_2_O_5_-28MgO-20K_2_O-5SO_3_ (labelled 37-5S).
- Zn-containing glasses:
  39SiO_2_-8P_2_O_5_-23MgO-20K_2_O-5ZnO-5SO_3_ (labelled as 39-5Zn);
  38SiO_2_-9P_2_O_5_-23MgO-20K_2_O-5ZnO-5SO_3_ (labelled as 38-5Zn);
  37SiO_2_-10P_2_O_5_-23MgO-20K_2_O-5ZnO-5SO_3_ (labelled as 37-5Zn).

Glass batches were prepared from high-purity analytical reagents: SiO_2_, (NH_4_)_2_HPO_4_, MgO, K_2_CO_3_, ZnO (for the second series) and K_2_SO_4_. To ensure complete decomposition of phosphates and sulfates while maintaining sulfur incorporation as sulfide (S^2−^), activated carbon was added in an amount that exceeded the stoichiometric requirement for K_2_SO_4_ reduction. This generated strongly reducing local conditions during melting, promoting the formation of mixed-anion (O^2−^/S^2−^) environments typical of reduced oxysulfide glasses.

### 2.2. Analysis of Chemical Composition

The chemical compositions of the synthesized glasses were quantified by X-ray fluorescence spectrometry (XRF) using a Thermo Scientific ARL Advant’XP spectrometer (Thermo Fisher Scientific, Waltham, MA, USA), with results normalized to 100%. The nominal compositions (mol%) and the experimentally determined values by XRF are presented in Table 1. Sample labels correspond to the molar proportions of their constituent oxides.

### 2.3. Thermal Analysis by Differential Scanning Calorimetry (DSC)

The thermal properties of the glasses were evaluated using a STA 449 F3 Jupiter (NETZSCH, Selb, Germany) instrument operating in DSC heat-flux mode. The key DSC parameters were as follows:

- Heating rate: 10 °C·min^−1^;
- Atmosphere: Synthetic air (40 mL·min^−1^);
- Sample mass: ~20 mg;
- Particle size: 0.1–0.3 mm.

Al_2_O_3_ was used as reference material. Glass transition temperatures (T_g_) and heat-capacity changes (ΔC_p_), the onset of the temperature of the first crystallization peak (T_c,o_) and the peak temperature of the first crystallization effect (T_c,p_) were determined using the NETZSCH Proteus Thermal Analysis software (version 8.03, NETZSCH-Gerätebau GmbH, Selb, Germany), with T_g_ defined as the midpoint of the transition step. The thermal stability of the glasses was assessed using the processing window ΔT = T_c,o_ − T_g_ [18].

### 2.4. Devitrification Treatment and X-Ray Diffraction (XRD)

For selected glasses, particles with sizes in the range of 0.1–0.3 mm were separated and subjected to isothermal heat treatment for 24 h at temperatures corresponding to the crystallization events identified by DSC. Phase identification of the devitrified materials was performed by X-ray diffraction using an X’Pert Pro Empyrean diffractometer (PANalytical, Malvern, UK) equipped with Cu Kα radiation (λ = 1.5406 Å). The diffraction patterns were collected in an appropriate 2θ range with a step size and a count time sufficient to ensure reliable phase identification.

Quantitative phase analysis was performed using the Rietveld refinement method. The refinements were performed using the HighScore Plus software (version 3.0e, Malvern Panalytical, Almelo, The Netherlands), employing crystallographic models obtained from the ICSD databases. The background, scale factors, lattice parameters, peak profile parameters, and phase fractions were iteratively refined until convergence was achieved. The phase fractions reported in this work correspond to the weight percentages obtained from the final refined models.

### 2.5. Electrical Characterization

Electrochemical impedance spectroscopy (EIS) measurements were conducted on polished samples equipped with silver electrodes using a GWInstek RLC meter (Good Will Instrument Co., Ltd., Taiwan, China). The experiments were performed over a broad temperature range, from room temperature up to 600 °C, and across frequencies spanning 100 Hz to 10 MHz, enabling the determination of both real and imaginary components of the electrical impedance and electric modulus. Temperature stability and control with an accuracy of ±0.1 °C were ensured by a programmable furnace.

Solid-State Impedance Spectroscopy (ss-IS) was used to study the electrical properties of selected samples below 300 °C. Gold electrodes were sputtered onto both sides of the bulk disks using a Sputter Coater SC7620 (Quorum Technologies, East Sussex, UK). Complex impedance was measured using an impedance analyser (Novocontrol Alpha-AN Dielectric Spectrometer, Novocontrol Technologies GmbH & Co. KG, Montabaur, Germany) in wide frequency (0.06 Hz to 1 MHz) and temperature (30 °C to 240 °C, step 30 °C) range. The temperature was controlled to an accuracy of ±0.2 K.

### 2.6. X-Ray Absorption Spectroscopy (XAS)

X-ray Absorption Spectroscopy (XAS) measurements were performed on devitrified 37-5S glass to investigate the local coordination environments of magnesium and potassium. XAS measurements of potassium and magnesium K-edge were performed ex situ on samples after isothermal heat treatment, i.e., after completion of devitrification, and therefore reflect the local structural state of the glass in its thermally treated form rather than real-time structural evolution during heating. Magnesium K-edge XAS spectra were collected at the PEEM/XAS beamline of the Solaris National Synchrotron Radiation Centre (Kraków, Poland) [19], using Total Electron Yield (TEY) detection at room temperature. This bending-magnet beamline provides soft X-ray radiation and is equipped with a plane-grating monochromator with an energy resolving power (E/ΔE) greater than 4000. The powdered sample were mounted on carbon tape for measurements. Complementary potassium K-edge XAS spectra of the same devitrified glass were acquired at the Elettra Synchrotron XAFS beamline (Sincrotrone Trieste S.C.p.A., Trieste, Italy) [20] in fluorescence mode using a fixed-exit Si(111) double-crystal monochromator and a silicon drift detector. All measurements were performed at room temperature under vacuum conditions. The collected spectra were processed, normalized, and analyzed using the Athena software (Version 0.9.26, Demeter package), available at https://bruceravel.github.io/demeter/, accessed on 15 April 2024 [21].

## 3. Results and Discussion

### 3.1. Chemical Composition and Sulfur Retention

**Figure 1:**
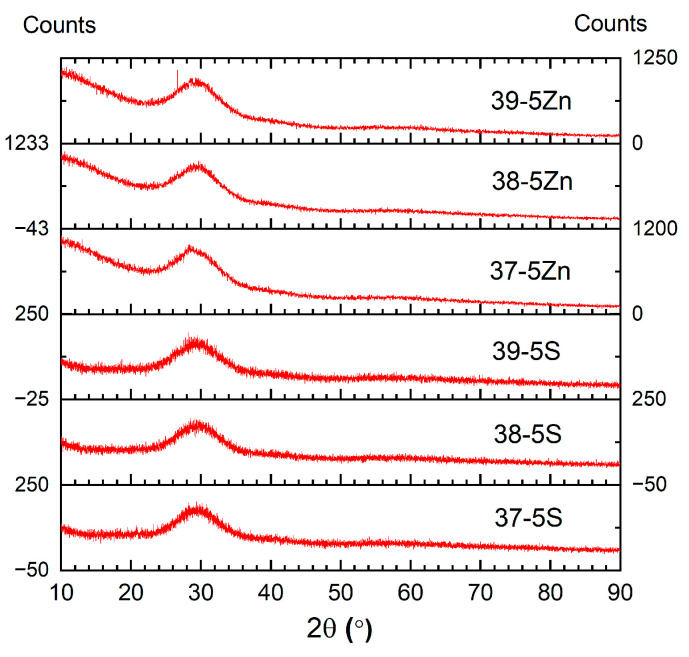
X-ray diffraction (XRD) patterns of the as-prepared glass samples (37-5S, 38-5S, 39-5S, 37-5Zn, 38-5Zn, and 39-5Zn), showing a broad diffuse halo characteristic of amorphous materials.

All synthesized glasses appeared visually homogeneous and exhibited a very dark burgundy coloration. Their amorphous nature was confirmed by X-ray diffraction, which revealed only a broad diffuse halo without any sharp Bragg reflections (Figure 1). Minor differences in coloration were observed between the Zn-free and Zn-containing glasses, suggesting compositional or structural variations induced by Zn addition. The chemical compositions determined by XRF, together with the nominal batch compositions for comparison, are summarized in Table 1.

XRF analysis revealed that partial substitution of MgO by ZnO led to a higher sulfur content retained in the glass structure. Glasses containing ZnO consistently exhibited higher measured SO_3_ contents than their Zn-free counterparts. This enhanced sulfur retention is accompanied by a reduction in the measured P_2_O_5_ content and by a pronounced loss of ZnO during melting.

In the Zn-free glass series, the SiO_2_ content decreases systematically from 43.7 to 40.2 mol%, while P_2_O_5_ increases from 3.8 to 6.1 mol% across the 39-5S to 37-5S compositions, reflecting the intended substitution of SiO_2_ by P_2_O_5_ network formers. The MgO content remains close to the nominal 28 mol%, confirming its role as the dominant alkaline-earth modifier. In all Zn-free glasses, the measured SO_3_ contents are slightly below the nominal value of 5 mol%, which is consistent with partial sulfur volatilization under reducing melting conditions [22].

In contrast, the Zn-containing glasses exhibit markedly lower ZnO concentrations than the nominal values, indicating substantial zinc loss during melting. This behavior is consistent with the high volatility of zinc under reducing conditions, where ZnO can be partially reduced to metallic Zn and subsequently evaporate. Thermodynamic and kinetic studies have demonstrated that ZnO reduction and zinc volatilization are strongly promoted at elevated temperatures in carbon-rich or reducing environments, particularly in multicomponent silicate melts [15,23,24]. The reduced ZnO concentrations measured by XRF therefore indicate that a considerable fraction of zinc was lost during melting rather than incorporated into the final glass structure.

XRF analysis also revealed the presence of several minor oxide components at concentrations typically below 1–2 mol%, in addition to the major glass-forming and modifying oxides. These minor constituents originate from raw-material impurities, crucible corrosion, and high-temperature processing effects. In particular, the presence of Al_2_O_3_ is attributed to partial dissolution of alumina crucibles during melting, with slightly higher Al_2_O_3_ contents observed in Zn-containing glasses. While Al_2_O_3_ incorporation can influence glass structure and crystallization behavior, its concentration in the present compositions remains sufficiently low that it does not obscure the intended compositional trends or affect the interpretation of the main thermal, structural, and electrical results [24,25].

### 3.2. Thermal Behavior of the Glasses (DSC)

**Figure 2:**
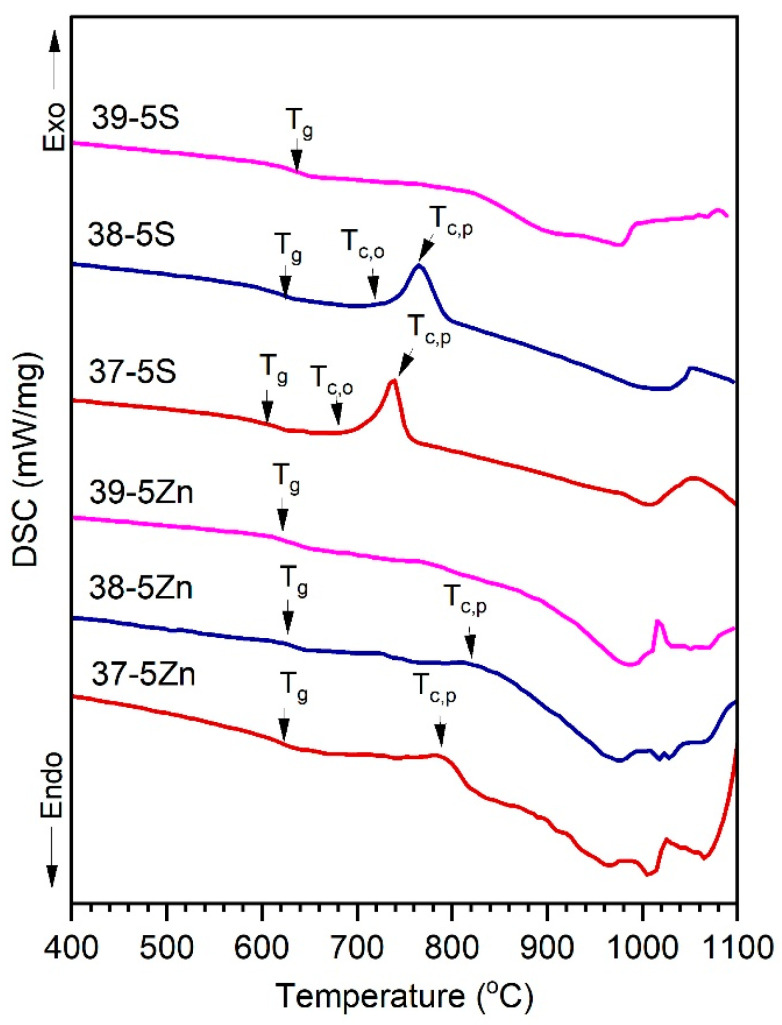
DSC profiles of the glasses without ZnO and with ZnO substitution.

The DSC curves of the Zn-free and Zn-containing glasses are shown in Figure 2, and the characteristic thermal parameters are summarized in Table 2. All compositions exhibit a distinct endothermic step associated with the glass transition. In the Zn-free series, the glass transition temperature (T_g_) decreases systematically from 626 to 597 °C with increasing P_2_O_5_. This behavior is characteristic of silicate–phosphate glasses and reflects progressive network depolymerization caused by the incorporation of phosphate units at the cost of silicate units and the associated increase in non-bridging oxygens coordinated to modifier cations [12,26]. Similar reductions in T_g_ with increasing phosphate concentration have been widely reported for multicomponent oxide glasses containing alkaline and alkaline-earth modifiers [12]. In contrast, the Zn-containing glasses exhibit a different T_g_ response to increasing P_2_O_5_ content. Although the lowest T_g_ is observed for the 39-5Zn composition, T_g_ increases with further phosphate addition. This reversal of the T_g_–P_2_O_5_ trend indicates a change in the structural role of phosphate units in the presence of Zn^2+^. Zinc is known to act as an intermediate oxide in silicate and phosphate glasses, forming more directional Zn–O bonds and participating in intermediate or mixed network-forming structural units that locally reinforce the network and counteract depolymerization effects [27,28].

The crystallization behavior also differs markedly between the two series. The Zn-free glasses 38-5S and 37-5S display sharp and well-defined crystallization exotherms, indicative of relatively high atomic mobility and diffusion-controlled crystal growth, as commonly observed in modifier-rich glass networks [29]. In contrast, the corresponding Zn-containing glasses exhibit broadened and weak crystallization features that are shifted to higher temperatures. Such suppression and broadening of crystallization exotherms are characteristic of glasses with increased network rigidity and reduced long-range diffusion, as previously reported for Zn-modified oxide and oxysulfide glasses [30].

The processing window (ΔT) calculated from the DSC data confirms that Zn-containing glasses possess enhanced kinetic stability and a reduced tendency toward crystallization compared to their Zn-free counterparts. The change in heat capacity at the glass transition (ΔC_p_) does not show a strictly monotonic compositional dependence; however, the generally lower ΔC_p_ values observed in the Zn-containing glasses indicate reduced configurational entropy and restricted local structural rearrangements near T_g_, consistent with the thermodynamic interpretation of the glass transition in rigid glass networks [30,31]. At higher temperatures, both glass series exhibit endothermic effects associated with melting or thermal decomposition. The upward shift in these effects observed for the Zn-containing glasses reflects the higher cohesive energy of Zn-containing structural units compared to Mg- and K-modified environments, which requires additional thermal energy to disrupt the glass network [24].

The observed reduction in configurational heat capacity (ΔCp), broadening and weakening of crystallization exotherms, and increase in melting-related endothermic temperatures with increasing ZnO content can be attributed to the intermediate role of Zn^2+^ in the glass network [27,28]. Zinc is known to form relatively strong and directional Zn–O bonds and to participate in mixed or intermediate structural units in silicate–phosphate glasses, which locally reinforce the glass structure and reduce the number of accessible configurational states near the glass transition [27,28]. This increased network rigidity limits long-range atomic diffusion and slows crystallization kinetics, leading to broadened and weaker crystallization exotherms shifted to higher temperatures [18]. At higher temperatures, the enhanced cohesive energy associated with Zn-containing structural units requires additional thermal energy to disrupt the network, resulting in elevated melting or decomposition temperatures [24]. Overall, the DSC results demonstrate that partial substitution of MgO by ZnO enhances the thermal stability of silicate–phosphate oxysulfide glasses by increasing network rigidity and slowing crystallization kinetics, while exerting only a moderate influence on the glass transition temperature.

### 3.3. Phase Composition of Devitrified Glasses (XRD)

Upon thermal treatment, glasses may undergo devitrification, as crystallization is thermodynamically favored due to the reduction in free energy associated with the transformation from a disordered amorphous structure to an ordered crystalline phase [1,2].

**Figure 3:**
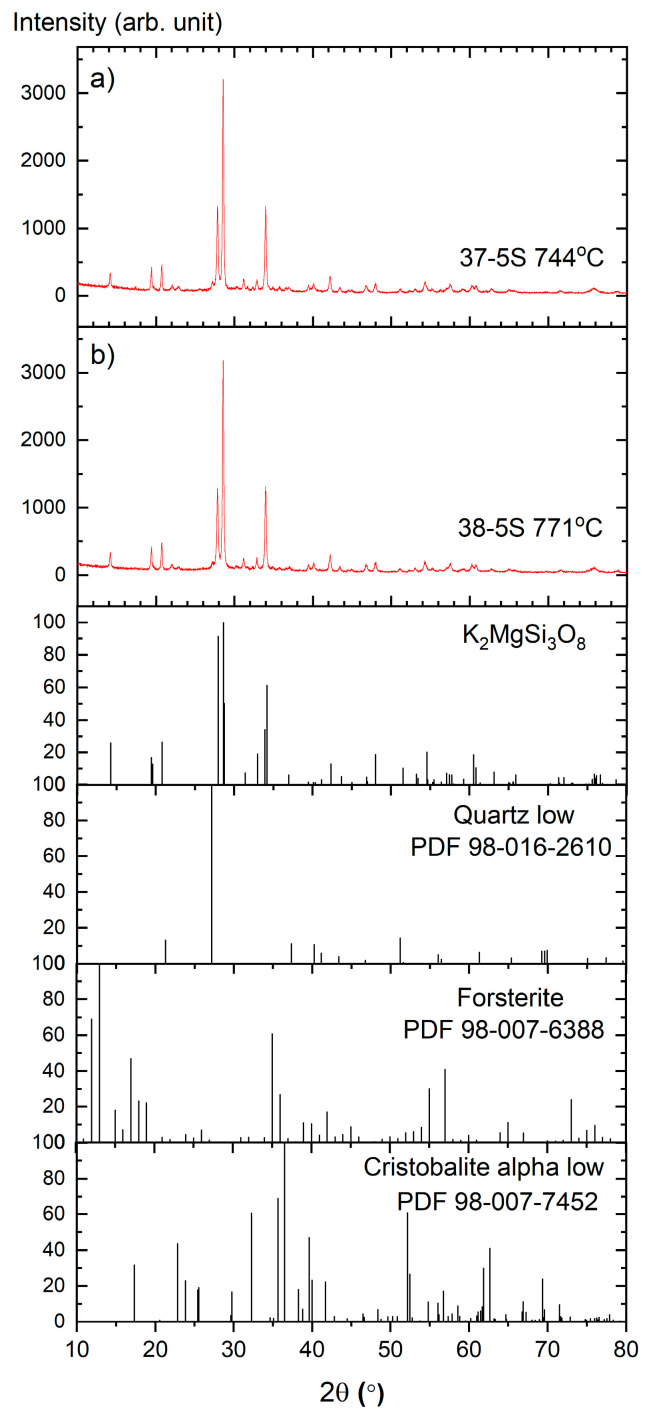
XRD patterns and Rietveld refinements of Zn-free glasses: (**a**) 37-5S heat-treated at 744 °C for 24 h and (**b**) 38-5S heat-treated at 771 °C for 24 h. The vertical tick marks indicate the Bragg reflections of the identified crystalline phases.

Figure 3 presents the X-ray diffraction (XRD) patterns of the Zn-free glasses after isothermal heat treatment, together with the corresponding Rietveld refinements. The 37-5S glass was annealed at 744 °C for 24 h (Figure 3a), while the 38-5S glass was treated at 771 °C for 24 h (Figure 3b). These temperatures were selected based on the crystallization events identified in the DSC curves (Figure 1). The good agreement between the experimental diffraction patterns and the calculated profiles confirms the reliability of the phase identification and quantitative analysis.

Rietveld refinement revealed dipotassium magnesium trisilicate, K_2_MgSi_3_O_8_, as the dominant crystalline phase in both devitrified samples, accounting for 85.4% in the 37-5S glass and 83.7% in the 38-5S glass. Forsterite, Mg_2_SiO_4_ (ICDD PDF 98-007-6388), was identified as a secondary crystalline phase, with phase fractions of 10.9% and 12.5% for the 37-5S and 38-5S samples, respectively. Minor crystalline phases include low-temperature α-cristobalite, SiO_2_ (ICDD PDF 98-007-7452), present at 1.6% in the 37-5S glass and 1.8% in the 38-5S glass, as well as low-temperature quartz, SiO_2_ (ICDD PDF 98-016-2610), accounting for 1.9% and 2.1% in the respective samples. No sulfur-containing crystalline phases were detected within the detection limits of the XRD measurements.

The observed phase assemblage suggests that, upon devitrification, modifier cations are not uniformly distributed within the silicate–phosphate network but tend to segregate into localized regions or clusters. Such structural heterogeneity may lead to the formation of preferential pathways that facilitate ionic transport, in agreement with previous reports on mixed-modifier glass systems [32].

The DSC curves of Zn-containing glasses exhibit weaker and broader crystallization effects shifted to higher temperatures, indicating reduced crystallization kinetics; therefore, devitrification was not achieved for these compositions under the heat-treatment conditions applied in this study.

### 3.4. Local Structure from XAS

#### 3.4.1. Potassium K-Edge XAS of the 37-5S Glass Devitrificate

**Figure 4:**
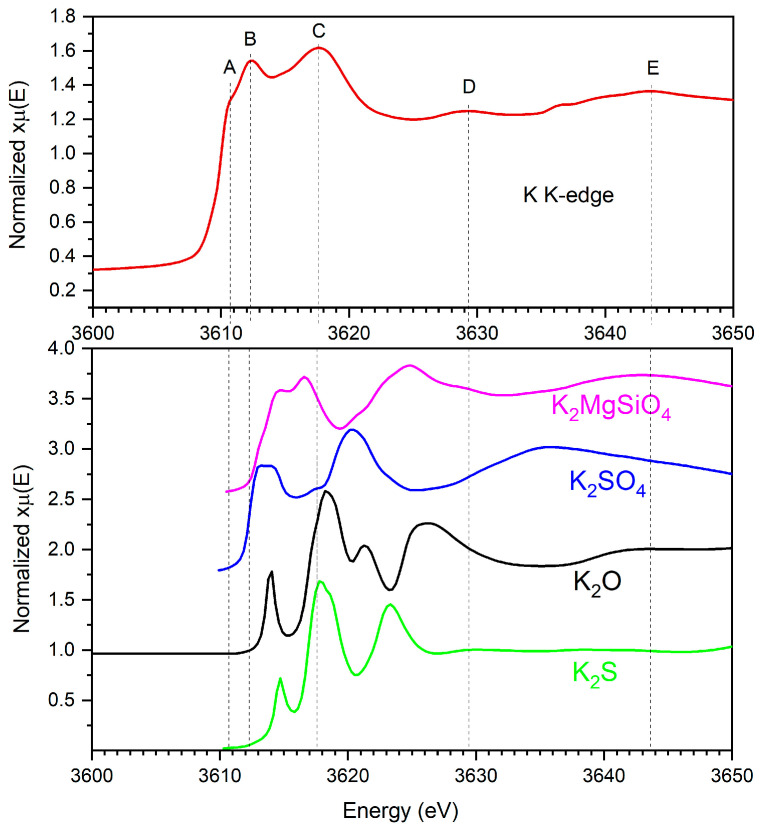
Potassium K-edge X-ray absorption spectra of the 37-5S glass devitrificate compared with reference spectra of selected potassium compounds (K_2_O, K_2_S, K_2_SO_4_, and K_2_MgSiO_4_) [33]. In the spectrum of the 37-5S glass devitrificate, the letters A–E indicate characteristic spectral features: A and B correspond to pre-edge and edge features, C to the main absorption peak (white line), D to the post-edge shoulder, and E to extended XAFS oscillations. The reference spectra are shown for qualitative comparison of spectral features and energy positions; no quantitative assignment of oxide or sulfide coordination is implied.

Potassium K-edge X-ray absorption spectra were collected ex situ for the Zn-free sulfur-containing glass (37-5S) after isothermal heat treatment at 744 °C for 24 h. The normalized K-edge XAS spectrum is shown in Figure 4 together with reference spectra of selected potassium compounds taken from the [33]. The absorption edge is located at approximately 3608 eV (feature A in Figure 4) and is followed by two pronounced resonances centered at about 3612 eV (feature B) and 3618 eV (feature C). A weak shoulder is observed close to the edge, marking the onset of the electronic transition.

As demonstrated by the reference spectra of crystalline potassium compounds such as K_2_O, K_2_S, K_2_SO_4_, and K_2_MgSiO_4_ (Figure 4), a principal resonance near 3612 eV is a common feature of K^+^-containing materials and is generally assigned to the dipole-allowed 1s → 4p transition, while a second feature around 3618–3619 eV arises from higher-energy final states and multiple-scattering contributions influenced by the surrounding coordination environment [34]. The positions of these resonances in the 37-5S glass devitrificate closely match those of the reference compounds, confirming that potassium remains in the +1 oxidation state after heat treatment. A slight shift in the resonances toward lower energy (≈0.3 eV) relative to purely oxide references is consistent with a more reduced local environment, as expected for sulfur-containing glasses.

Compared with the reference spectra, the glass spectrum exhibits broader features and reduced post-edge oscillations (features D and E in Figure 4), indicating a distribution of local potassium environments rather than a single well-defined crystalline coordination. The relative sharpening and separation of the two main resonances after devitrification suggest an increase in local and medium-range order around K^+^ ions; however, the absence of pronounced extended fine-structure oscillations beyond ~3625 eV implies that this ordering remains confined to nanometric domains rather than extended crystalline phases.

Because the glass was synthesized under reducing conditions, sulfur is expected to be predominantly present in reduced form. Nevertheless, the K-edge XAS data alone do not allow a quantitative distinction between oxide- and sulfide-based coordination environments. The reference spectra are therefore used exclusively for qualitative benchmarking of spectral features and energy positions, without explicit assignment of specific K–O or K–S bonding configurations. The exact sulfur speciation cannot be determined without complementary S-edge measurements, and the possible presence of polysulfide units, as reported for alkali-containing sulfide glass systems, cannot be excluded [35].

In summary, the potassium K-edge XAS results indicate that devitrification of the 37-5S glass is accompanied by an increase in short- and medium-range structural ordering around K^+^ ions while preserving their monovalent oxidation state. These observations are consistent with the crystallization behavior inferred from DSC measurements and support a scenario in which potassium participates in network rearrangement during devitrification without forming long-range ordered potassium-rich crystalline phases.

#### 3.4.2. Magnesium K-Edge XAS of the 37-5S Glass Devitrificate

**Figure 5:**
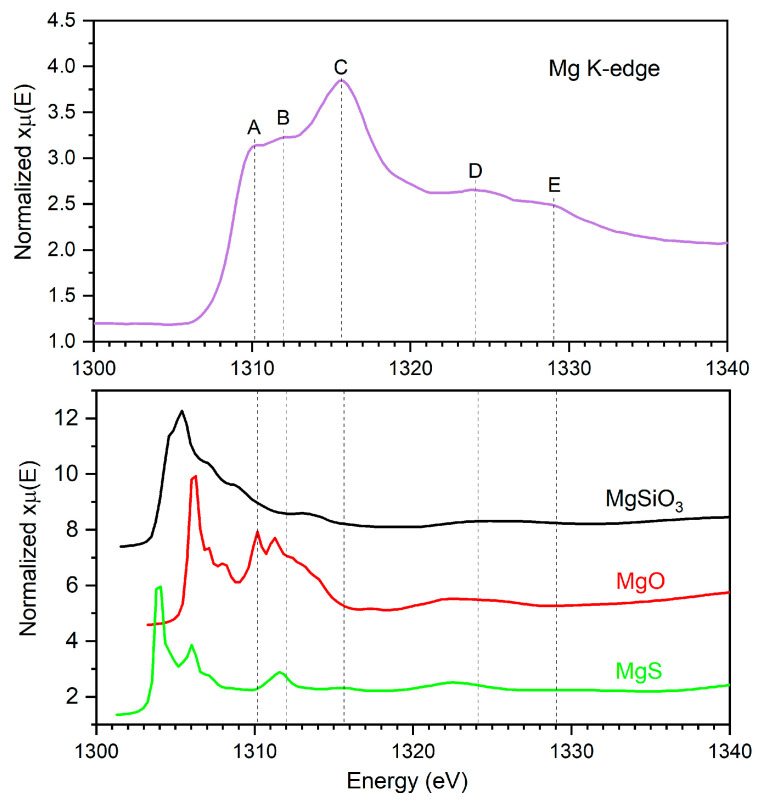
Magnesium K-edge X-ray absorption spectra of the 37-5S glass devitrificate compared with reference spectra of MgO, MgS, and MgSiO_3_ from the literature [33]. In the spectrum of the 37-5S glass devitrificate, the letters A–E indicate characteristic spectral features: A, absorption edge onset; B and C, main near-edge resonance features (white-line region); D and E, post-edge features associated with extended XAFS oscillations. The reference spectra are included to qualitatively benchmark the spectral features of the glass against oxide- and sulfide-based environments, without implying a quantitative determination of Mg–O or Mg–S coordination.

Magnesium K-edge X-ray absorption spectra were collected ex situ for the Zn-free sulfur-containing glass (37-5S) after isothermal heat treatment at 744 °C for 24 h. The normalized Mg K-edge XAS spectrum is shown in Figure 5, together with reference spectra of MgO, MgS, and MgSiO_3_ taken from the literature [33]. The absorption edge onset at approximately 1304 eV (feature A in Figure 5) is characteristic of Mg^2+^ and confirms that magnesium remains in a divalent oxidation state after thermal treatment under reducing conditions [36].

The near-edge region exhibits a structured spectral shape with several overlapping features between approximately 1310 and 1320 eV. Comparison with the reference compounds indicates that these features are sensitive to the local coordination environment of Mg^2+^. In contrast to the sharper resonances observed for crystalline MgO and MgSiO_3_, the glass spectrum shows broadened features, reflecting a distribution of local Mg environments typical of partially ordered or nanostructured materials.

No significant shift in the absorption edge relative to oxide-based reference spectra is observed, suggesting that magnesium does not undergo redox changes during devitrification. The spectral broadening and reduced post-edge modulation compared with crystalline references indicate limited medium-range order and the formation of nanometric ordered domains embedded within a residual glassy matrix.

The reference spectra of MgO and MgS are included for qualitative benchmarking only. While similarities with both oxide- and sulfide-based reference spectra can be observed, the Mg K-edge XAS data alone do not allow a quantitative distinction between Mg–O and Mg–S coordination. Therefore, the interpretation is restricted to qualitative assessment of changes in local ordering and coordination heterogeneity around Mg^2+^ ions following devitrification.

Two broad, low-intensity features observed at higher energies (D and E) mark the onset of post-edge oscillations. Their limited amplitude and rapid damping indicate that medium-range order around Mg^2+^ remains weak, consistent with the presence of nanometric ordered regions embedded in a residual glassy matrix rather than the formation of extended crystalline Mg-rich phases. Overall, these spectral characteristics point to increased local ordering combined with significant coordination heterogeneity around magnesium after devitrification.

In summary, the Mg K-edge XAS results indicate that devitrification of the 37-5S glass is accompanied by an increase in short-range structural ordering around Mg^2+^ while preserving its divalent oxidation state. The persistence of broadened spectral features suggests that magnesium remains distributed within heterogeneous local environments rather than forming extended, well-ordered Mg-rich crystalline phases, in agreement with the XRD and DSC results.

### 3.5. Electrical and Dielectric Properties

**Figure 6:**
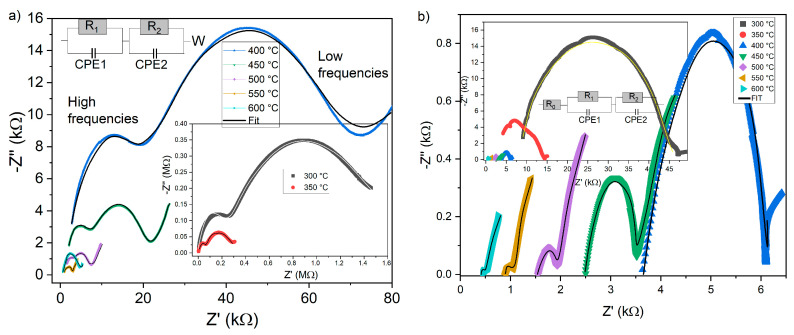
Nyquist diagrams for 38-5S and 38-5Zn glasses at selected temperatures. Inset shows corresponding equivalent circuit. (**a**) 38-5S; (**b**) 38-5Zn.

Examining how impedance varies with frequency and temperature offers important insight into changes in the intrinsic (bulk) resistance of the glass samples. The impedance is a complex function, where the real component Z′ reflects the resistive behavior, and the imaginary component Z″ represents the capacitive response of the material. In electrochemical impedance spectroscopy (EIS) studies of glass, parameters such as bulk resistance, relaxation time, and activation energy can be extracted from the Nyquist plot by fitting equivalent electrical circuits. The bulk resistance is obtained from the high-frequency intercept on the real axis, the relaxation time from the characteristic frequency of the semicircle, and the activation energy from the temperature dependence of conductivity or relaxation time. The activation energy E_a_ from conductivity is related to long-range ion transport through the glass network, while the activation energy from relaxation time reflects localized ion hopping or short-range motion. Nyquist plots for 38-5S and 38-5Zn glasses are shown in Figure 6a and Figure 6b, respectively.

For glass 38-5S, the Nyquist plot contains two semicircular arcs and a Warburg element (Figure 6a). The high-frequency arc corresponds to bulk processes, whereas the low-frequency arc is associated with the electrode–glass interface At higher temperatures, the Warburg-type response becomes more evident in the low-frequency region, reflecting enhanced ionic diffusion at the glass–electrode interface. The bulk resistance is determined from the high-frequency intercept of the first semicircle with the Z′ axis, while the local minimum in Z″ between the two semicircles marks the transition between bulk and interfacial processes.

In contrast, the Zn-containing 38-5Zn glass (Figure 6b) exhibits a single, depressed semicircular arc without a detectable Warburg element. The absence of diffusion-controlled impedance suggests suppression of long-range ionic transport, consistent with restricted modifier mobility caused by the formation of stronger Zn–O/S structural units. This interpretation is in agreement with the reduced crystallization tendency observed by DSC [27]. The semicircles do not originate at the Z′ = 0 point but are shifted away from the origin, indicating the presence of a series resistance and/or unresolved high-frequency processes, while the depressed shape reflects non-Debye relaxation associated with structural heterogeneity of the glass.

**Figure 7:**
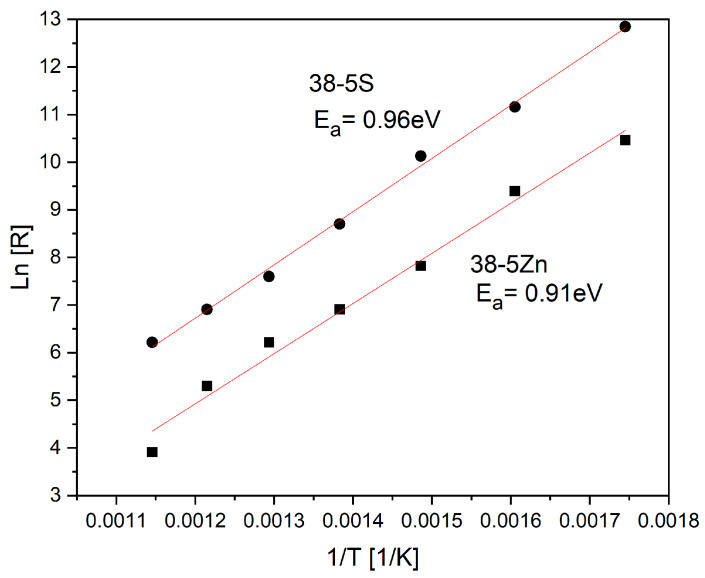
Arrhenius plot for 38-5S and 38-5Zn glass.

A suitable fitted EEC (Electrical Equivalent Circuit) model is essential for accurately capturing the electrical response of the different regions within the material. The fitted curves reproduce the experimental data closely. Each semicircle in the Nyquist diagram is modeled using a parallel R–C element. However, in both cases, the capacitor is replaced by a constant-phase element (CPE) to account for the depressed, non-ideal semicircles whose centers lie below the real axis. This behavior is characteristic of glassy and crystalline materials exhibiting non-Debye relaxation. In the classical Debye model, a single relaxation time generates a perfect semicircle in the complex impedance plane, with its center positioned directly on the real (Z′) axis. The semicircular arcs progressively contract with increasing temperature for both types of glasses. Elevated temperatures generate more space-charge carriers, enhance electron hopping, and thus confirm the semiconducting nature of the materials. The 38-5Zn glass shows significantly lower impedance values (both capacity and resistance) compared to the 38-5S glass. Figure 7 shows the dependence of the electrical resistance of 38-5Zn and 38-5S glasses on temperature. Sample 38-5S exhibits significantly higher resistance values than 38-5Zn. The slope of the temperature dependence is constant, which suggests that only one electrical conduction mechanism is present in this temperature region.

All fitted data were validated using the Kramers–Kronig (K–K) test, confirming the physical consistency, linearity, and stability of the measured impedance spectra. The selected equivalent circuit models show R^2^ values show deviations below 10%.

The activation energy E_a_ of electrical resistivity was performed using the Arrhenius equation:

(1) $$k=A\cdot e^{\frac{-E_{a}}{RT}}$$

where *A* is the pre-exponential constant, E_a_ represents the activation energy, *T* is the absolute temperature, and *R* is gas constant [37]

The activation energy E_a_ was found to be 0.96 eV and 0.91eV for 38-5S and 38-5Zn, respectively, in the investigated temperature range and it was found to be consistent with the observed trend for conductivity of the glass materials. Based on the relatively high activation energy (~1 eV), ionic conduction is identified as the dominant transport mechanism in these materials.

**Figure 8:**
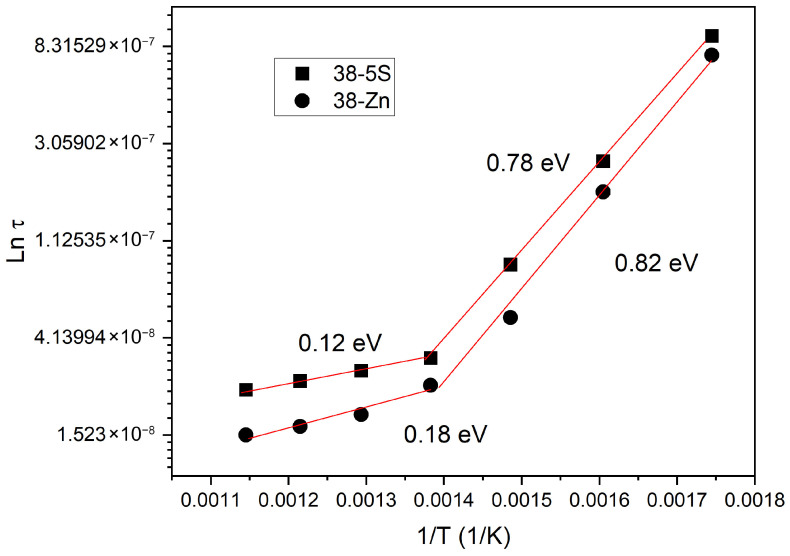
Relaxation time versus inverse temperature for 38-5S and 38-5Zn glasses. *E_a_* is the activation energy.

To determine the relaxation time at each temperature, the frequency at which −Z″ reaches its maximum *f_max_* was identified and present in Figure 8. The relaxation time τ is calculated from this relaxation frequency using the relationship [38]:

(2) $$\tau =1/(2\pi f_{max})$$

Figure 8 presents the variation in the relaxation time τ as a function of inverse temperature (1/*T*). The analysis was carried out using a modified Arrhenius relation [37,39]:

(3) $$\tau =\tau_{0}exp(\frac{E_{a}}{K_{B}T}),$$

where *τ*_0_ is the pre-exponential constant.

The activation energy associated with the relaxation time ($E_{a,rel}$) was estimated to be 0.78 eV and 0.82 eV for the 38-5S and 38-5Zn glasses, respectively, in the lower temperature region (≈300–450 °C). These values are characteristic of thermally activated long-range ionic hopping processes, where mobile modifier ions migrate between energetically favorable sites within the disordered glass network. Such activation energies are commonly reported for ion transport in oxide glasses and indicate that relaxation and electrical conduction are governed by the same charge-carrier dynamics in this temperature regime. In contrast, at higher temperatures (≈450–600 °C), the activation energy decreases significantly to 0.12 eV for 38-5S and 0.18 eV for 38-5Zn. This reduction suggests a transition to relaxation mechanisms associated with lower energy barriers, such as localized ionic motions, short-range hopping, or secondary (β-type) relaxation processes. At elevated temperatures, increased thermal energy allows charge carriers to access a broader distribution of pathways with reduced barriers, resulting in enhanced mobility and lower apparent activation energies.

Additionally, the electrical conductivity for 39-5S and 39-5Zn samples was studied at even lower temperatures, spanning from RT to 240 °C, see Figure S1 in Supplemental Information. Similarly, to the 38-5S and 38-5Zn, a linear temperature dependence of DC conductivity in this temperature range was observed. The corresponding activation energies were estimated to be 0.77 eV and 0.73 eV for the 39-5S and 39-5Zn glasses, respectively. This is in line with thermally activated long-range ionic hopping processes within the disordered glass network.

The observed temperature-dependent change in activation energy reflects the disordered nature of the glass network and the coexistence of multiple relaxation mechanisms. The close correlation between relaxation behavior and electrical conductivity confirms that ionic transport plays a dominant role in both processes across the investigated temperature range.

#### 3.5.1. Electrical Capacitance

**Figure 9:**
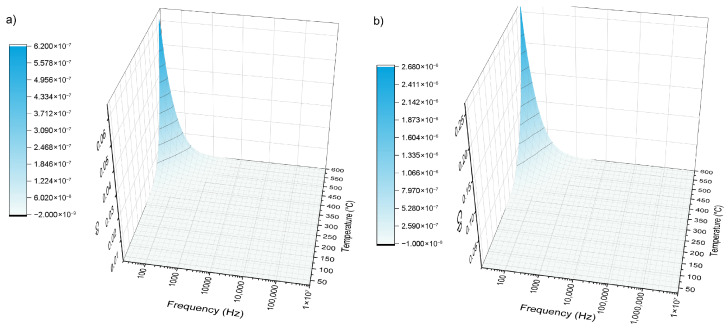
The temperature and frequency- evolution of electrical capacitance *C_p_* for 38-5S and 38-5Zn glasses. (**a**) 38-5S; (**b**) 38-5Zn.

Measurements of the electrical capacitance, *C_p_*, as a function of both frequency and temperature were carried out, and results are presented in Figure 9. The responses of the two samples were similar, exhibiting a noticeable increase in capacitance only at the lowest frequencies, below 1 kHz, and at elevated temperatures above 400 °C. This behavior indicates that the dielectric response in this regime is strongly frequency- and temperature-dependent, with significant contributions arising primarily under conditions where slow polarization mechanisms or thermally activated processes become dominant. The sample 38-5Zn achieves higher values of *Cp* than 38-5S. Such trends are characteristic of materials in which dipolar relaxation or defect- and modifier-related polarization mechanisms are pronounced at low frequencies and high temperatures.

#### 3.5.2. Evolution of the Dielectric Losses

**Figure 10:**
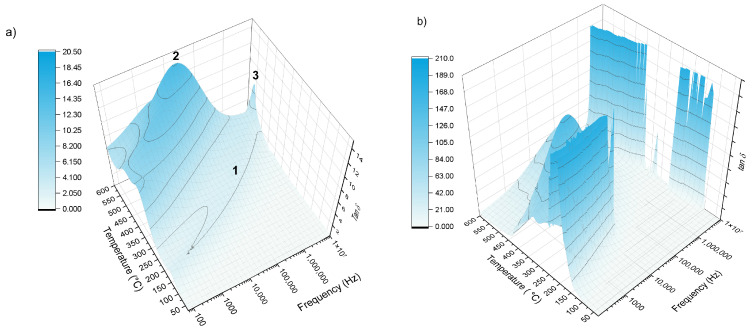
The evolution of tan d in function of temperature and frequency for (**a**) 38-5S and (**b**) 38-5Zn.

The dielectric losses tan δ (Figure 10) spectrum represents the ratio of the imaginary to the real part of the permittivity (ε″/ε′) and thus describes how effectively the material dissipates electrical energy [40]. The observed dependence of tan δ on temperature and frequency arises from the interplay between ionic conduction and dipolar relaxation mechanisms inherent to disordered glass networks [40]. Generally, for glasses, this curve typically rises with temperature, exhibits a peak at a characteristic relaxation frequency, and then declines at higher frequencies [41]. The low-frequency side of the peak is governed mainly by ionic conduction, while the maximum occurs when the field oscillation rate matches the characteristic reorientation time of dipolar groups within the glass structure, allowing maximum energy absorption. Beyond this relaxation frequency, at higher frequencies, the structural units of the glass cannot reorient fast enough to follow the applied field, leading to a continued decrease in dielectric loss. This behavior reflects the intrinsic relaxation dynamics of disordered glass networks and their limited ability to respond to very rapid electrical perturbations. Both samples exhibit distinct tan δ behaviors as functions of frequency and temperature, and in both cases the dielectric response is relatively complex.

For the first glass composition 38-5S (38SiO_2_–9P_2_O_5_–28MgO–20K_2_O–5SO_3_), three characteristic regions can be identified in the tan δ spectra. Between 150–500 °C, the tan δ curves display a broad local maximum (marked as “1” in Figure 10a) that resembles a single relaxation wave: extending across the entire measured frequency range. As the temperature increases, this maximum systematically shifts to higher frequencies, reaching maximum approximately 10 MHz at 500 °C. This behavior is typical of thermally activated dipolar or ionic relaxation, where increased temperature enhances the mobility of network modifiers (e.g., K^+^, Mg^2+^) and structural units within the mixed SiO_2_–P_2_O_5_ network [12,42]. A second region shows global asymmetric maximum (“2”), more pronounced feature appears at around 550 °C, where the tan δ reaches a global maximum of about tan δ = 18 near 500 kHz. The peak shape is notably asymmetric, with the high-frequency side dropping more steeply than the low-frequency side. Such asymmetry often indicates the presence of distributed relaxation times—a signature of structural disorder and the coexistence of multiple relaxation mechanisms (ionic hopping, dipole reorientation, and localized structural rearrangements) within the glass [42,43].

The final region exhibits a sharp peak (“3”) was present in the high-temperature and high-frequency region. At still higher temperatures and frequencies, the spectra reveal a sharp, well-defined maximum. This sharp relaxation is commonly associated with fast ionic polarization processes or glass-transition-related structural dynamics, where the glass network becomes sufficiently softened to allow rapid local motion. In mixed-oxide phosphate–silicate glasses, this can involve accelerated mobility of modifier cations (particularly K^+^) or rapid reorganization of SO_3_-containing structural groups [44]. For the Zn-doped 38SiO_2_–9P_2_O_5_–28MgO–20K_2_O–5SO_3_ (38-5Zn) glass, the tan δ response within the same temperature and frequency range differs substantially from that of the undoped sample, showing overall much higher dielectric loss. Although both glasses share general features such as a mid-frequency maximum between approximately 50 kHz and 1 MHz above 450 °C and another maximum in the highest-frequency region at elevated temperatures, the Zn-containing glass displays two particularly strong relaxation peaks at lower temperatures that are absent in the original composition.

The first pronounced maximum appears between about 150 and 250 °C at around 1 kHz and reaches remarkably high tan δ values, indicating that Zn^2+^ incorporation strongly modifies the mobility of ions and enhances dipolar polarization. Literature on Zn-modified silicate and phosphate glasses reports that ZnO has been reported to act as an intermediate oxide capable of modifying network connectivity and enhancing localized polarization and hopping processes and dielectric losses [18]. The second strong maximum, observed between roughly 250 and 370 °C near the same 1 kHz frequency, suggests an additional thermally activated relaxation process involving more substantial structural rearrangements or cooperative dynamics of mixed silicate–phosphate units. Previous studies have shown that Zn-containing glasses can form Zn–O–Si and Zn–O–P linkages, creating localized polar structures that respond slowly to the external field and produce distinct relaxation modes [12,27]. These structural effects collectively increase the polarizability of the glass, introduce a more heterogeneous distribution of mobile ions, and generate additional dipolar and ionic relaxation pathways, resulting in a significantly more complex and pronounced tan δ behavior compared with the undoped glass. Although Zn-containing glasses exhibit higher dielectric losses, this does not imply enhanced long-range ionic conductivity. The increased tan δ reflects intensified localized polarization and short-range hopping processes within structurally heterogeneous regions of the glass network associated with Zn^2+^ incorporation, while extended ion diffusion remains hindered [40,41].

The electrical conductivity mechanisms in the investigated glasses are strongly governed by the nature of modifier cations and their bonding environments within the mixed oxysulfide network. In the Zn-free glasses, charge transport is dominated by thermally activated ionic hopping, primarily involving K^+^ ions and, to a lesser extent, Mg^2+^ ions. The presence of relatively ionic Mg-O, Mg-S, and K-S bonds creates flexible modifier-rich regions and continuous percolation pathways that facilitate long-range ionic diffusion. This mechanism is supported by the appearance of two semicircular arcs and a Warburg diffusion element in the Nyquist plots, as well as by broad low-frequency tan δ maxima characteristic of ionic conduction in disordered glass networks [45,46].

In contrast, substitution of MgO by ZnO markedly alters the conduction mechanism. Zinc behaves as an intermediate oxide that locally increases network rigidity through more directional metal–oxygen interactions and mixed network-forming structural units. These effects reduce free volume and disrupt continuous ionic pathways, thereby suppressing long-range diffusion. As a result, the Zn-containing glasses exhibit a single depressed semicircle without a Warburg contribution, indicating that electrical transport is dominated by localized hopping and dipolar polarization processes rather than extended ionic motion [12,26]. Despite their higher dielectric losses, the Zn-containing glasses display increased resistivity, demonstrating that enhanced tan δ arises from localized polarization within Zn-rich structural domains rather than improved macroscopic conductivity [17].

Overall, Mg-rich glasses favor diffusion-controlled ionic transport, whereas Zn incorporation induces a transition toward polarization-dominated, short-range charge dynamics. This mechanistic distinction is consistent with the DSC results, which show suppressed crystallization in Zn-containing compositions. Taken together, XAS, DSC, and EI spectroscopy consistently demonstrate that Mg promotes structural ordering that facilitates crystallization through ionic Mg-O/S bonding, whereas Zn^2+^ incorporation stabilizes the glass network by increasing local rigidity and suppressing long-range ionic transport, thereby enhancing thermal stability and promoting localized polarization dynamics.

#### 3.5.3. The Evolution of Dielectric Constant

**Figure 11:**
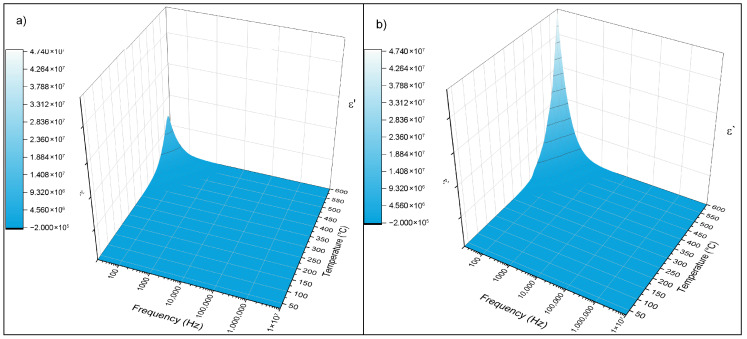
The evolution of ε′ as a function of temperature and frequency for 38-5S and 38-5Zn. (**a**) 38-5S; (**b**) 38-5Zn.

The real part of the dielectric constant ε′ as a function of frequency and temperature for both glass compositions exhibits a clear increase with rising temperature, particularly in the low-frequency region (Figure 11). This behavior is typical of ionically conducting glasses and is associated with thermally activated hopping of mobile charge carriers, predominantly K^+^ ions, which contribute to space-charge and interfacial polarization at low frequencies. As temperature increases, the enhanced mobility of these charge carriers leads to a pronounced rise in ε′, especially below 1 kHz.

With increasing frequency, ε′ decreases steadily for both compositions. At higher frequencies, charge carriers and dipolar units are unable to reorient rapidly enough to follow the alternating electric field, resulting in a reduced contribution to polarization and a progressive decline in the dielectric constant. This frequency-dependent behavior reflects the limited response time of mobile ions and dipoles in the disordered glass network.

Compared with the Zn-free glass, the Zn-containing composition exhibits systematically lower e′ values over the investigated frequency and temperature ranges. This trend indicates reduced space-charge polarization and diminished long-range ionic mobility upon ZnO incorporation. The decrease in e′ is attributed to increased network rigidity and reduced free volume, which hinder the formation of continuous alkali-ion migration pathways and limit the contribution of mobile charge carriers to dielectric polarization.

Overall, impedance spectroscopy demonstrates that Zn incorporation fundamentally alters the dielectric response of the glasses. While Zn-containing compositions exhibit enhanced dielectric loss and multiple relaxation processes due to increased localized polarization and short-range hopping, long-range ionic diffusion is suppressed, as evidenced by the absence of Warburg-type behavior in the impedance spectra. These electrical characteristics are consistent with the DSC results, which indicate enhanced thermal stability and suppressed crystallization in Zn-containing glasses. Together, the electrical and thermal data confirm that Zn^2+^ acts as an intermediate oxide that stabilizes the glass network by restricting extended ionic transport while promoting heterogeneous local polarization dynamics.

## 4. Conclusions

Reduced silicate–phosphate oxysulfide glasses in the SiO_2_–P_2_O_5_–K_2_O–MgO–SO_3_ system, with and without partial substitution of MgO by ZnO, were systematically investigated using DSC, XRD, XAS, and impedance spectroscopy. All compositions exhibited a well-defined glass transition and non-Debye electrical relaxation, confirming their structurally heterogeneous nature.

DSC results show that increasing ZnO content decreases the configurational heat capacity at the glass transition, suppresses crystallization exotherms, and shifts crystallization events to higher temperatures, indicating enhanced thermal stability and reduced crystallization tendency. XRD analysis confirms that Zn-free glasses readily devitrify into potassium–magnesium silicate phases, whereas crystallization is strongly suppressed in Zn-containing compositions.

K and Mg K-edge XAS reveal that magnesium promotes short-range structural ordering and facilitates devitrification in Zn-free glasses, while Zn-containing glasses remain more structurally disordered after heat treatment. These differences are consistent with the contrasting crystallization behavior observed by DSC and XRD.

Impedance spectroscopy demonstrates that ZnO substitution leads to higher activation energies and reduced AC conductivity, reflecting restricted alkali-ion mobility. Although Zn-containing glasses exhibit increased dielectric losses and additional relaxation processes, these effects are attributed to localized polarization and short-range ionic hopping rather than enhanced long-range ionic transport.

Overall, partial substitution of MgO with ZnO stabilizes reduced silicate–phosphate oxysulfide glass networks against crystallization while significantly modifying their electrical response. These findings provide clear structure–property relationships relevant to the design of thermally stable functional oxysulfide glasses.

The dielectric constant is closely related to the AC conductivity. Zinc substitution alters ion mobility and modifies the short-range order. The higher activation energy is associated with microstructural features and an increased number of defects, as well as enhanced local disorder.

## Figures and Tables

**Table 1:** XRF-derived chemical compositions of the glasses (mol%), with nominal batch compositions given in parentheses for comparison. Values reported as <DL indicate concentrations below the detection limit of the XRF method.

Comp.	39-5S	38-5S	37-5S	39-5Zn	38-5Zn	37-5Zn
SiO_2_	43.68 (39)	41.28 (38)	40.16 (37)	46.74 (39)	46.26 (38)	44.05 (37)
P_2_O_5_	3.80 (8)	5.28 (9)	6.13 (10)	3.01 (8)	3.79 (9)	4.70 (10)
K_2_O	16.96 (20)	18.14 (20)	18.52 (20)	18.98 (20)	19.38 (20)	18.29 (20)
MgO	30.63 (28)	30.06 (28)	29.99 (28)	23.24 (23)	24.59 (23)	23.71 (23)
SO_3_	2.99 (5)	2.36 (5)	2.14 (5)	4.61 (5)	4.68 (5)	4.70 (5)
ZnO	<DL	<DL	<DL	0.13 (5)	0.06 (5)	0.16 (5)
Al_2_O_3_	0.69	1.15	1.28	2.04	0.98	2.80
Na_2_O	1.04	1.31	1.32	1.02	<DL	1.10
CaO	0.12	0.13	0.13	0.13	0.16	0.33
TiO_2_	0.03	0.03	0.04	0.02	0.03	0.03
Fe_2_O_3_	0.02	0.02	0.02	0.02	0.02	0.03
CuO	<DL	<DL	<DL	0.01	<DL	0.01
Rb_2_O	0.01	0.01	0.01	<DL	<DL	<DL
SrO	<DL	<DL	<DL	<DL	<DL	<DL
ZrO_2_	<DL	0.21	0.23	0.01	0.02	<DL
Cr_2_O_3_	<DL	<DL	<DL	<DL	<DL	<DL
NiO	<DL	<DL	<DL	<DL	<DL	0.01
Sm_2_O_3_	<DL	<DL	<DL	<DL	<DL	0.02
Cl	0.02	0.02	0.02	0.04	0.03	<DL

**Table 2:** Characteristic thermal parameters of the investigated glasses determined from DSC measurements: glass transition temperature (T_g_), onset temperature of the first crystallization peak (T_c,o_), peak temperature of the first crystallization event (T_c,p_), processing window (ΔT), and change in heat capacity at T_g_ (ΔC_p_). Note: “–” indicates that no crystallization event was detected within the investigated temperature range, and therefore T_c,o_, T_c,p_, and ΔT could not be determined.

Glass Name	T_g_/°C	T_c,o_/°C	T_c,p_/°C	ΔT/K	ΔC_p_/J/(g·K)
39-5S	626	–	–	–	0.163
38-5S	615	740	765	125	0.200
37-5S	597	697	738	100	0.225
39-5Zn	590	–	–	–	0.220
38-5Zn	638	829	838	191	0.113
37-5Zn	614	763	791	149	0.215

## Data Availability

The original contributions presented in this study are included in the article/Supplementary Materials. Further inquiries can be directed to the corresponding authors.

## Supplementary Materials

The following supporting information can be downloaded at: https://www.mdpi.com/article/10.3390/ma19040734/s1, Figure S1: Arrhenius plot of DC electrical conductivity (log σ_DC) as a function of inverse temperature (1000 T^−1^) for 39-5S and 39-5Zn glasses. The solid lines represent linear fits used to determine the activation energies for DC conduction.
