# Supplementary material for: Stabilization of Mixed-Anion (O2−/S2−) Networks in ZnO-Substituted Silicate–Phosphate Oxysulfide Glasses: Linking Cation–Sulfide Bonding to Thermal and Dielectric Properties
Source: Materials (Basel). 2026 Feb 13;19(4):734. doi: 10.3390/ma19040734 (PMC12942353; doi:10.3390/ma19040734)
Supplement: Supplementary file 1 [file materials-19-00734-s001.zip › materials-4113819-supplementary.pdf]

Supplementary Materials

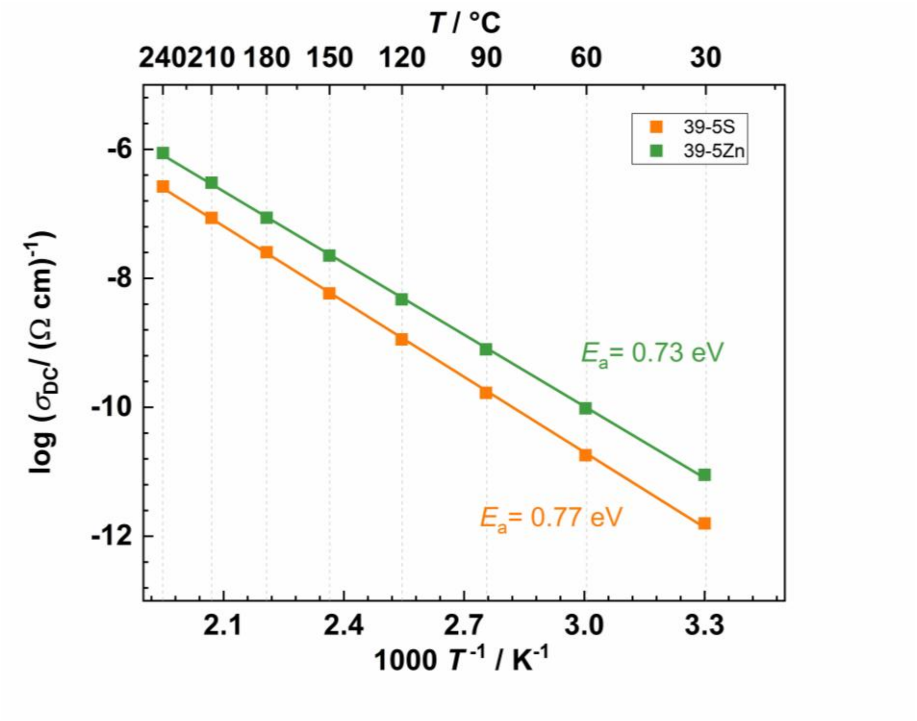

Figure. S1. Arrhenius plot of DC electrical conductivity (log σ_DC) as a function of inverse temperature (1000 T^{-1}) for 39-5S and 39-5Zn glasses. The solid lines represent linear fits used to determine the activation energies for DC conduction.
